# Saracatinib inhibits necroptosis and ameliorates psoriatic inflammation by targeting MLKL

**DOI:** 10.1038/s41419-024-06514-y

**Published:** 2024-02-08

**Authors:** Jingyi Li, Xingfeng Liu, Yuanyuan Liu, Fangmin Huang, Jiankun Liang, Yingying Lin, Fen Hu, Jianting Feng, Zeteng Han, Yushi Chen, Xuan Chen, Qiaofa Lin, Lanqin Wu, Lisheng Li

**Affiliations:** 1 The School of Basic Medical Sciences, Fujian Medical University, Fuzhou, China; 2 Key Laboratory of Ministry of Education for Gastrointestinal Cancer, Fujian Medical University, 1 Xueyuan Road, Minhou Fuzhou, China

**Keywords:** Necroptosis, Psoriasis

## Abstract

Necroptosis is a kind of programmed cell death that causes the release of damage-associated molecular patterns and inflammatory disease including skin inflammation. Activation of receptor-interacting serine/threonine kinase 1 (RIPK1), RIPK3, and mixed lineage kinase domain-like protein (MLKL) is the hallmark of tumour necrosis factor α (TNF)-induced necroptosis. Here, we screened a small-molecule compound library and found that saracatinib inhibited TNF-induced necroptosis. By targeting MLKL, Saracatinib interfered with the phosphorylation, translocation, and oligomerization of MLKL induced by TNF. Consistently, mutation of the saracatinib-binding site of MLKL reduced the inhibitory effect of saracatinib on TNF-induced necroptosis. In an imiquimod (IMQ)-induced psoriasis mouse model, saracatinib effectively blocked MLKL phosphorylation and inflammatory responses in vivo. Taken together, these findings indicate that saracatinib inhibits necroptosis by targeting MLKL, providing a potential therapeutic approach for skin inflammation-related diseases such as psoriasis.

## Introduction

Necroptosis is a type of programmed cell death with necrotic characteristics, including cell swelling, plasma membrane rupture, and the release of damage-associated molecular patterns (DAMPs), which trigger inflammation [1, 2]. Among the types of necroptosis resulting from various stimuli, tumour necrosis factor α (TNF)-induced necroptosis is the most extensively studied. Upon binding of TNF, TNF receptor 1 (TNFR1) recruits downstream effectors, including TNFR1-associated death domain (TRADD), TNFR-associated factor 2 (TRAF2), receptor-interacting protein kinase 1 (RIPK1) and inhibitor of κB kinase (IKK) complex, to form signalling complex I [3]. Complex I regulates cell survival and proliferation through downstream NF-κB and MAPK signalling pathways. Deubiquitination of RIPK1 by CYLD promotes the recruitment of multiple proteins, including FADD (Fas-associated via death domain), Caspase-8, and receptor-interacting protein kinase 3 (RIPK3), by RIPK1 [4, 5]. When caspase-8 is absent or inhibited, RIPK1 and RIPK3 form necrosomes, which lead to the activation and autophosphorylation of RIPK3 [6–8]. Autophosphorylation of RIPK3 is needed for the recruitment of mixed lineage kinase domain-like pseudokinase (MLKL) [9–12]. The interaction of RIPK3 and MLKL leads to the phosphorylation of MLKL, which in turn favours the oligomerization and membrane targeting of MLKL to execute necroptosis [13–16]. In addition to RIPK3, multiple proteins, including TAM (Tyro3, Axl, Mer) kinases [17], inositol phosphate kinases [18], and heat shock protein 90 (HSP90) [19], have been shown to modulate the oligomerization and membrane targeting of MLKL. However, the detailed mechanism underlying the oligomerization and membrane targeting of MLKL still needs further investigation.

Necroptosis plays important roles in the pathology of multiple diseases, including atherosclerosis, autoimmune diseases, acute kidney injury, bowel inflammation, neurodegenerative diseases, and acute pancreatitis [20]. Necroptosis has also been implicated in skin inflammation. Epidermis-specific RIPK1 knockout or mutation of the RIP homotypic interaction motif (RHIM) of RIPK1 in keratinocytes leads to skin inflammation due to ZBP1/RIPK3/MLKL-mediated necroptosis [21–24]. Moreover, inhibition of necroptosis prevents Stevens-Johnson syndrome (SJS) and toxic epidermal necrolysis (TEN)-like inflammatory responses in a mouse model of SJS/TEN [25]. Similarly, necroptosis inhibitors such as Nec-1s strongly prevent inflammatory responses in a mouse psoriasis model induced by imiquimod (IMQ) [26]. Importantly, the RIPK1 inhibitor GSK2982772 was found to reduce epidermal thickness and infiltration of the epidermis and dermis by CD3 + T cells in a phase IIa clinical trial of plaque-type psoriasis [27]. Thus, inhibition of necroptosis by small-molecule compounds would be effective in the treatment of skin inflammation-related diseases.

To identify a new inhibitor of necroptosis, we screened a small-molecule compound library and found that saracatinib effectively inhibited TNF-induced necroptosis in different cell types. Saracatinib inhibited necroptosis induced by dimerization/oligomerization of the TNFR1 death domain, RIPK1, or RIPK3 but not the MLKL N-terminal domain (aa 1-190). Saracatinib did not inhibit TNF-induced phosphorylation of RIPK1 or RIPK3. In contrast, saracatinib interfered with the phosphorylation, translocation, and oligomerization of MLKL. Interestingly, saracatinib was able to bind to MLKL according to molecular docking simulation and increased the thermal stability of MLKL proteins, which suggested that MLKL is a potential target of saracatinib. Furthermore, mutation of Gln343Ala in MLKL, which is one of saracatinib-binding sites of MLKL, reduced the inhibitory effect of saracatinib on TNF-induced necroptosis. In an IMQ-induced psoriasis mouse model, saracatinib effectively blocked MLKL phosphorylation and inflammatory responses in mice. Thus, our work reveals that saracatinib inhibited necroptosis and ameliorated IMQ-induced psoriasis in mice by targeting MLKL.

## Results

### Saracatinib is a novel necroptosis inhibitor identified by a cell screen

**Fig. 1:**
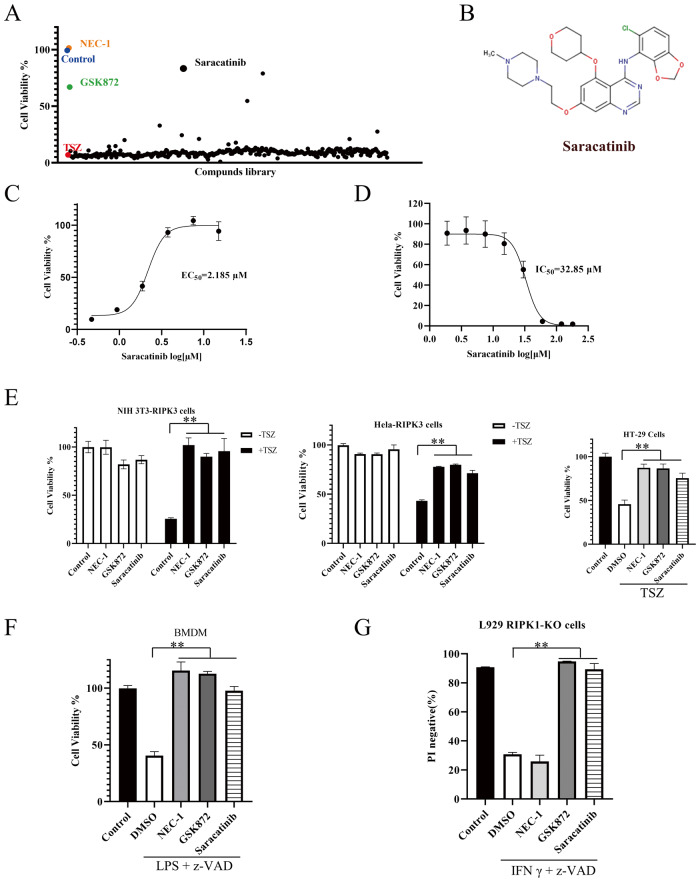
Saracatinib inhibited necroptosis in different cell lines. **A** L929 cells were pretreated with individual compound (10 μM) for 1 h following treatment with TNF (10 ng/ml), Smac mimetic (SM-164) (0.1 μM) and pan-caspase inhibitor z-VAD (10 μM) (TSZ) to induce necroptosis. Cell viabilities were determined with Cell Counting Kit-8 (CCK8) method and normalized to control (DMSO only). NEC-1 (20 μM) and GSK872 (10 μM) were chose as positive control. The screen data for each compound was singlicate. **B** Chemical structure of saracatinib cited from TargetMol. **C** L929 cells were pretreated with saracatinib at different concentrations for 1 h prior to treatment with TSZ. Cell viabilities were determined by CCK8 method (*n* = 3). **D** L929 cells were treated with saracatinib at different concentrations for 24 h. Then cell viabilities were determined by CCK8 method *(n* = 6). **E** NIH-3T3-RIPK3 cells, Hela-RIPK3 cells, and HT-29 cells were pretreated with NEC-1 (20 μM), GSK872 (10 μM) or saracatinib (7.5 μM) for 1 h prior to treatment with TSZ. Cell viabilities were determined by CCK8 method (*n* = 6 for NIH-3T3-RIPK3 cells, *n* = 3 for Hela-RIPK3 cells, *n* = 8 for HT-29 cells). **F** Bone marrow-derived macrophages (BMDM) were pretreated with NEC-1 (20 μM), GSK872 (10 μM) or saracatinib (15 μM) for 1 h prior to treatment with LPS + z-VAD. Cell viabilities were determined by CellTiter-Glo Luminescent Cell Viability Assay (*n* = 5). **G** L929-RIPK1 KO cells were seeded in 24-well plates and pretreated with NEC-1 (20 μM), GSK872 (10 μM) or saracatinib (15 μM) for 1 h prior to treatment with IFN-γ (20 ng/ul) and z-VAD (10 μM). Cells were harvested and incubated with propidium iodide (PI) (*n* = 3). PI negative cells were analyzed by flow cytometry. **: *p* < 0.01. Data are represented as mean ± SD. See also Fig. S1.

To identify novel necroptotic inhibitors, we evaluated compounds in an in-house library for their ability to inhibit necroptosis induced by TNF-α, the Smac mimetic SM-164, and the pan-caspase inhibitor z-VAD (TSZ) in murine L929 cells. We found that saracatinib significantly inhibited TSZ-induced necroptosis (Fig. 1A, B). The ability of saracatinib to inhibit TNF-induced necroptosis was further confirmed by propidium iodide staining (Fig. S1A). Since saracatinib was the most effective compound in inhibiting TNF-induced necroptosis according to our screening. Moreover, Saracatinib has been used in multiple clinical trials such as Alzheimer’s disease, Idiopathic Pulmonary Fibrosis [28, 29]. It would be interest to repurpose saracatinib as a necroptosis inhibitor. Then, we determined the half-maximal effective concentration (EC_50_) of saracatinib in inhibiting necroptosis and the half-maximal inhibitory concentration (IC_50_) of saracatinib. As shown in Fig. 1C, D, the EC_50_ and IC_50_ of saracatinib were 2.185 μM or 32.85 μM, respectively. Although saracatinib was cytotoxic at high concentrations, it inhibited necroptosis at more than 10-fold lower concentrations; thus, it might be possible to inhibit necroptosis with saracatinib without inducing cell toxicity.

To further confirm the role of saracatinib in regulating TNF-induced necroptosis, we tested whether saracatinib could inhibit necroptosis in murine NIH-3T3-RIPK3 cells, human HeLa-RIPK3 cells and HT-29 cells. As shown in Fig. 1E, saracatinib effectively inhibited necroptosis in these cell lines. Moreover, saracatinib also inhibited LPS-induced necroptosis in bone marrow-derived macrophages (BMDM) (Figs. 1F and S1B). Interferon-γ (IFN-γ) induced necroptosis in an RIPK1-independent, RIPK3- and MLKL-dependent manner [30, 31]. Consistently, the RIPK3 inhibitor GSK’872, but not the RIPK1 inhibitor NEC-1, sufficiently inhibited IFN-γ-induced necroptosis in RIPK1-KO cells (Figs. 1G and S1C). We found that saracatinib inhibited IFN-γ-induced necroptosis in a concentration-dependent manner (Figs. 1G and S1C), suggesting that saracatinib inhibited necroptosis downstream of RIPK1. Collectively, these data indicated that saracatinib efficiently blocked necroptosis.

### Saracatinib inhibits cell death caused by forced dimerization of TNFR1, RIPK1 and RIPK3

**Fig. 2:**
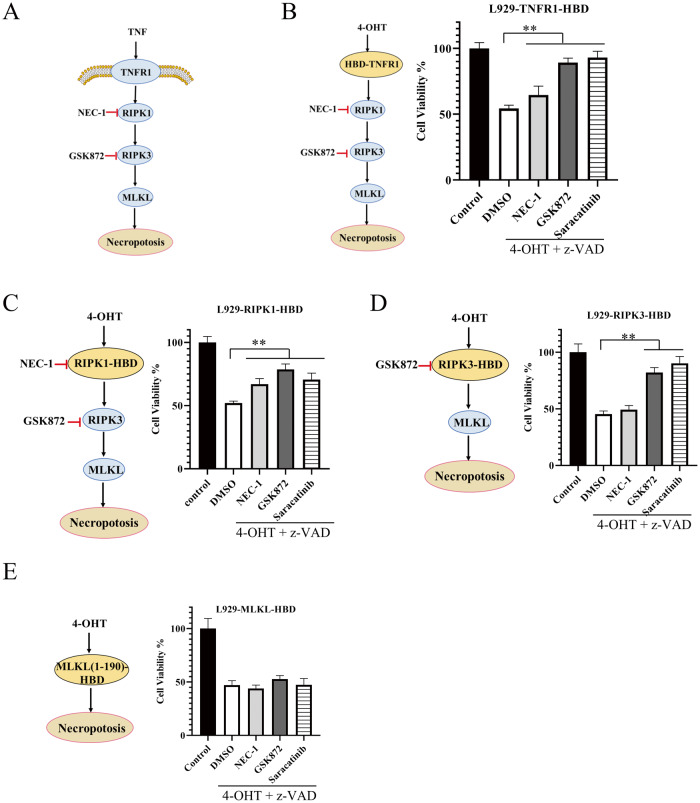
The role of saracatinib in Artificially induced dimerization/oligomerization of TNFR1-DD, RIPK1, RIPK3 and MLKL-1-190. **A** Brief description of TNF-induced necroptosis pathway. **B–E** Stable L929 cell line expressing TNFR1-DD-HBD (**B**), RIPK1-HBD (**C**), RIPK3-HBD (**D**) or MLKL-1-190-HBD (**E**) were pretreated with NEC-1 (20 μM), GSK872 (10 μM) or saracatinib (7.5 μM) for 1 h following treatment with 4-OHT and z-VAD. Then the cell viabilities were determined by CCK8 method (*n* = 6 for Fig. 2B–E). **: *p* < 0.01. Data are represented as mean ± SD. See also Fig. S2.

TNF-induced necroptosis is mediated by key proteins, including TNFR1/RIPK1/RIPK3/MLKL (Fig. 2A). Artificially induced dimerization/oligomerization of proteins in the necroptosis signalling pathway was able to induce necroptosis [32–34]. Then, we tested whether saracatinib could interfere with necroptosis induced by dimerization/oligomerization of the TNFR1-death domain (DD), RIPK1, RIPK3, and MLKL-N-terminal domain (1-190). All of these proteins were fused with the HBD domain (Fig. S2A–C) and were able to artificially form dimers/oligomers in response to the compound 4-OHT. Consistent with the role of RIPK1 and RIPK3 in the necroptosis signalling pathway, the RIPK1 inhibitor NEC-1 inhibited necroptosis induced by dimerization/oligomerization of TNFR1-DD or RIPK1 but not necroptosis induced by dimerization/oligomerization of RIPK3 or MLKL-1-190 (Fig. 2B–E). In contrast, the RIPK3 inhibitor GSK872 inhibited necroptosis induced by dimerization/oligomerization of TNFR1-DD, RIPK1 or RIPK3 but not necroptosis induced by dimerization/oligomerization of MLKL-1-190 (Fig. 2B–E). We found that saracatinib significantly inhibited necroptosis induced by dimerization/oligomerization of TNFR1, RIPK1 or RIPK3 but not necroptosis induced by dimerization/oligomerization of MLKL-1-190 (Fig. 2B–E), which indicated that saracatinib inhibited necroptosis through RIPK3 or its downstream signalling.

### Saracatinib inhibits necroptosis by targeting downstream RIPK3 signalling

**Fig. 3:**
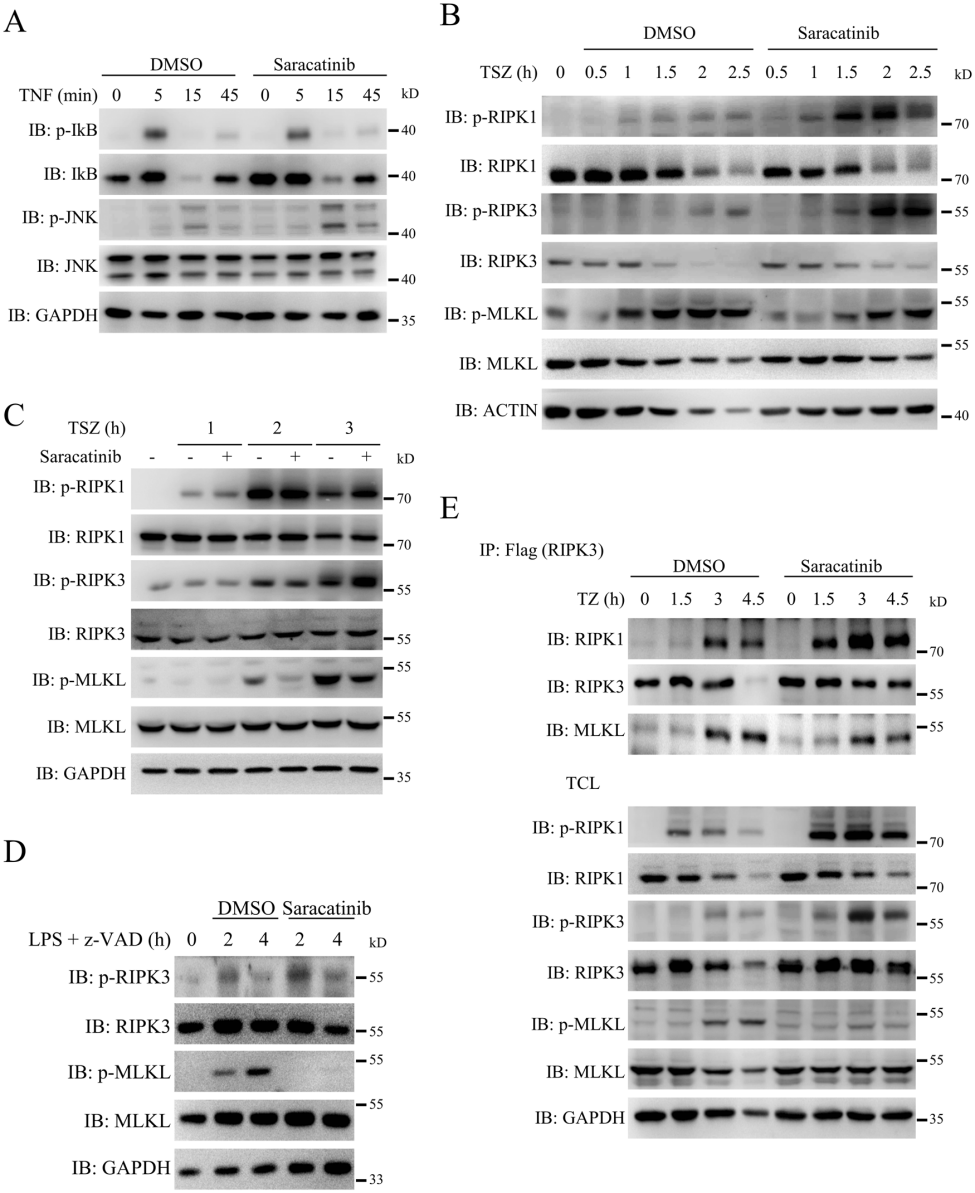
Saracatinib inhibited necroptosis through targeting downstream of RIPK3. **A** L929 cells were pretreated with DMSO or saracatinib (7.5 μM) for 1 h following treatment with TNF for indicated time. Then the cells were harvested and analyzed with the indicated antibodies (*n* = 3). **B** and **C** L929 (B) or HT-29 (**C**) cells were pretreated with DMSO or saracatinib (7.5 μM) for 1 h following treatment with TSZ for indicated time. Then the cells were harvested and analyzed with the indicated antibodies (*n* = 3). **D** BMDM cells were pretreated with DMSO or saracatinib (7.5 μM) for 1 h following treatment with LPS + z-VAD. Then the cells were harvested and analyzed with the indicated antibodies (*n* = 3). **E** Mouse RIPK3-flag reconstituted RIPK3-KO L929 cells were pretreated DMSO or saracatinib (7.5 μM) for 1 h following treatment with TNF + zVAD (TZ). Then the cells were harvested and immunoprecipitated with M2 (anti-flag) antibody. The total cell lysates (TCL) and the immunoprecipitates were immunoblotted with the indicated antibodies (*n* = 3). See also Fig. S3.

To further explore the mechanism by which saracatinib inhibited TNF-induced necroptosis, we tested the effect of saracatinib on the TNF signalling pathway. As shown in Figs. 3A and S3A, saracatinib did not inhibit TNF-induced phosphorylation of IκB and JNK, which indicated that saracatinib did not disrupt TNFR1 complex 1 signalling. Phosphorylation of RIPK1, RIPK3 and MLKL is the key event in the TNF-induced necroptosis signalling pathway. To our surprise, saracatinib increased TNF-induced autophosphorylation of RIPK1 and RIPK3 but attenuated the phosphorylation of MLKL in L929 cells (Figs. 3B and S3B), which indicated that saracatinib did not inhibit the kinase activity of RIPK1 and RIPK3. Consistently, saracatinib reduced phosphorylation of MLKL but not phosphorylation of RIPK1 or RIPK3 in HT-29 cells treated with TNF and in BMDM cells treated with LPS (Figs. 3C, D, S3C, D). The formation of necrosomes, which consist of RIPK1/RIPK3/MLKL, is a critical step in TNF-induced necroptosis [3, 9, 13]. We found that saracatinib attenuated the interaction of RIPK3 and MLKL but not the interaction of RIPK1 and RIPK3 (Figs. 3E and S3E). Since saracatinib inhibited necroptosis without interfering autophosphorylation of RIPK3 and the interaction of RIPK1 and RIPK3, we propose that saracatinib inhibited necroptosis by regulating downstream RIPK3 signalling.

### MLKL is a potential target of saracatinib

**Fig. 4:**
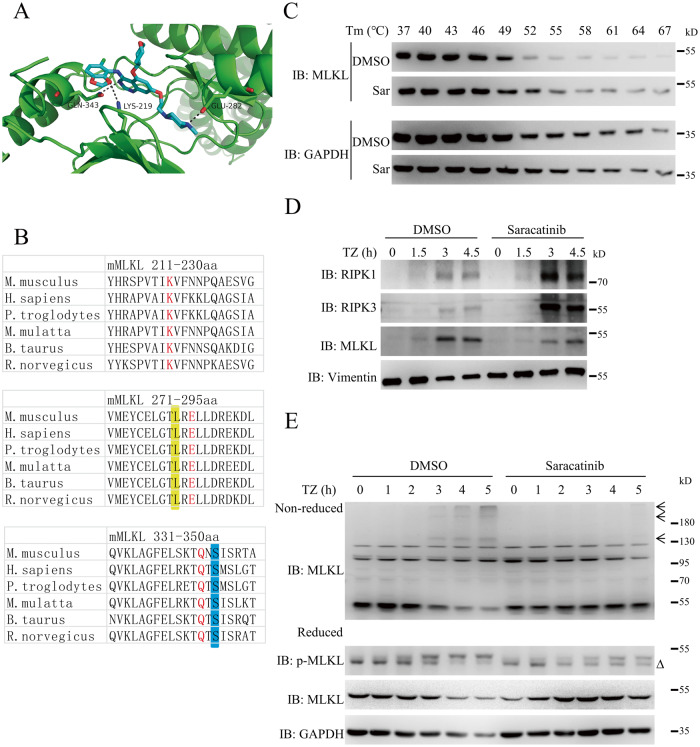
MLKL is a potential target of saracatinib. **A** Molecular Docking simulation result of saracatinib and MLKL using MLKL structure (PDB code: 4BTF). **B** Alignment of binding sites between saracatinib and MLKL from different species. Residue numbers referred to the mouse MLKL sequence. The binding sites between saracatinib and MLKL were highlighted in red. The orthologs of the residue mutated in human stomach cancer L291 (mouse L280) was highlighted in yellow. The phosphorylation site of mouse MLKL S345 was highlighted in blue. **C** The protein stability of MLKL were determent using CETSA assay method in L929 cells treated with DMSO or saracatinib (*n* = 3). **D** L929 cells were pretreated saracatinib (7.5 μM) or DMSO for 1 h following treatment with TZ for indicated time. Then the cells were harvested and lysed with Triton X-100 lysis buffer. The insoluble fractions were collected by centrifuge and analyzed with the indicated antibodies (*n* = 3). **E** L929 cells were pretreated saracatinib (7.5 μM) or DMSO for 1 h following treatment with TZ for indicated time. Then the each cell samples were divided into two parts and lysed with reduced SDS sample buffer (50 mM Tris-Hcl pH6.8, 2% SDS, 10% glycerin, 12.5 mM EDTA, 1% β-mercaptoethanol, 0.02% bromophenol blue) or non-reduced SDS sample buffer (50 mM Tris-Hcl pH6.8, 2% SDS, 10% glycerin, 12.5 mM EDTA, 0.02% bromophenol blue). Then the samples were analyzed with the indicated antibodies (*n* = 3). ∆: nonspecific band. The oligomerization of MLKL was marked with arrow. See also Fig. S4.

MLKL is the critical downstream target of RIPK3. MLKL has a kinase-like domain and was proposed to be a pseudokinase due to the lack of conserved catalytic residues that are crucial for phosphoryl transfer activity [35]. Since saracatinib is a kinase inhibitor, we proposed that saracatinib might target MLKL. The crystal structure of mouse MLKL has been reported (PDB code: 4BTF) [35]; therefore, we performing molecular docking analysis of saracatinib and MLKL. As shown in Fig. 4A, saracatinib formed hydrogen bonds with the Lys219, Glu282 and Gln343 residues of the MLKL kinase-like domain. Importantly, the Lys219, Glu282 and Gln343 residues of MLKL are highly conserved among different species (Fig. 4B). Upon binding to a compound, the thermal stability of a protein changes [36]. Based on this principle, the cellular thermal shift assay (CETSA) was used to investigate the interaction of compounds and their potential target proteins [36]. As shown in Figs. 4C and S4A, we found that treatment with saracatinib increased the thermal stability of MLKL, which further suggests MLKL is a potential target of saracatinib. Translocation of RIPK1/RIPK3/MLKL into detergent-insoluble fractions is important for necroptosis signal transduction [3, 9]. As shown in Figs. 4D and S4B, saracatinib promoted the translocation of RIPK1 and RIPK3 into the Triton X-100 insoluble fraction. In contrast, saracatinib specifically inhibited the translocation of MLKL, further suggesting that saracatinib inhibited necroptosis by MLKL. Activation of MLKL by RIPK3 leads to the oligomerization and plasma membrane translocation of MLKL and the execution of necroptosis [12, 16]. Then, we tested whether saracatinib influenced the oligomerization and plasma membrane translocation of MLKL. As shown in Figs. 4E and S4C, TNF-induced MLKL oligomerization was abolished by saracatinib. Moreover, saracatinib inhibited the plasma membrane translocation of MLKL (Fig. S5). Taken together, these results suggest that MLKL is a target of saracatinib.

### Gln343 of MLKL is involved in the inhibition of necroptosis by saracatinib

**Fig. 5:**
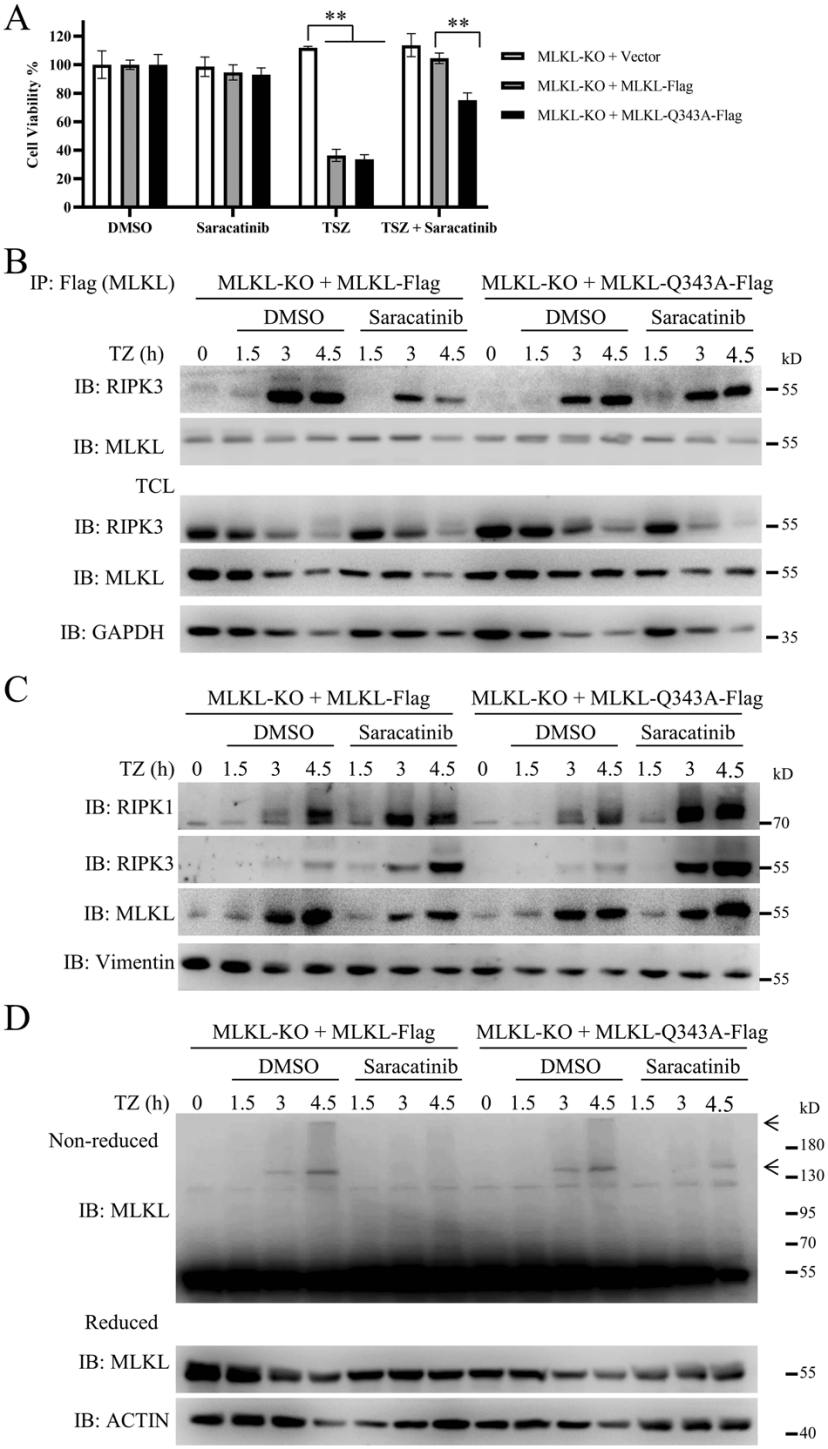
Gln343 of MLKL is involved in the inhibition of necroptosis by saracatinib. **A** MLKL-KO L929 cells were reconstituted with vector, MLKL-Flag or MLKL-Q343A-Flag. Then these cells were pretreated saracatinib (7.5 μM) or DMSO for 1 h following treatment with TSZ. Then the cell viabilities were determined by CCK8 method (*n* = 4). **B** MLKL-Flag or MLKL-Q343A-Flag reconstituted MLKL-KO L929 cells were pretreated saracatinib (7.5 μM) or DMSO for 1 h following treatment with TZ for indicated time. Then the cells were harvested and immunoprecipitated with M2 (anti-flag) antibody. The total cell lysates (TCL) and the immunoprecipitates were immunoblotted with the indicated antibodies (*n* = 3). **C** MLKL-Flag or MLKL-Q343A-Flag reconstituted MLKL-KO L929 cells were pretreated saracatinib (7.5 μM) or DMSO for 1 h following treatment with TZ for indicated time. Then the cells were harvested and lysed with Triton X-100 lysis buffer. The insoluble fractions were collected by centrifuge and analyzed with the indicated antibodies (*n* = 3). **D** MLKL-Flag or MLKL-Q343A-Flag reconstituted MLKL-KO L929 cells were pretreated saracatinib (7.5 μM) or DMSO for 1 h following treatment with TZ for indicated time. Then each cell samples were divided into two parts and lysed with reduced SDS sample buffer or nonreduced SDS sample buffer. Then the samples were analyzed with the indicated antibodies (*n* = 3). The oligomerization of MLKL was marked with arrow. **: *p* < 0.01. Data are represented as mean ± SD. See also Figs. S6 and S7.

According to crystal structure of MLKL (PDB code: 4BTF), the Q343 residue, which is in the atypical activation loop helix, interacts with the conventional ATP binding residue K219 through hydrogen bonding [35]. Overexpression of MLKL with mutation of Lys219Met (K219M) or Gln343Ala (Q343A) induces lytic cell death in mouse dermal fibroblasts (MDFs) [35]. Considering that saracatinib bound to the Lys219, Glu282 and Gln343 residues of MLKL, we evaluated whether saracatinib regulates necroptosis through these binding sites. L929 MLKL-KO cells were infected with lentivirus expressing wild-type MLKL, MLKL K219M, E282A or Q343A, respectively. For unknown reasons, we observed the expression of wild-type MLKL and MLKL-Q343A but not MLKL-K219M and MLKL-E282A in L929 MLKL-KO cells (Fig. S6A). We found that the expression of wild-type MLKL or MLKL-Q343A restored sensitivity to TNF-induced necroptosis in MLKL-KO L929 cells (Fig. S6B) [26]. Moreover, saracatinib significantly inhibited TNF-induced necroptosis in MLKL-KO L929 cells expressing wild-type MLKL (Fig. 5A). In contrast, the ability of saracatinib to inhibit necroptosis was reduced in MLKL-KO L929 cells expressing MLKL-Q343A compared with those expressing wild-type MLKL (Fig. 5A). Moreover, saracatinib inhibited the TNF-induced interaction of RIPK3 and MLKL, the translocation of MLKL into the Triton X-100 insoluble fraction, and the oligomerization of MLKL in MLKL-KO L929 cells expressing wild-type MLKL (Figs. 5B–D, S7A–D). In contrast, these effects were impaired in MLKL-KO L929 cells expressing MLKL-Q343A (Figs. 5B–D, S7A–D). Taken together, these results suggest that saracatinib inhibited TNF-induced necroptosis at least partly through the Gln343 residue of MLKL.

### Saracatinib alleviates IMQ-induced psoriasiform dermatitis

**Fig. 6:**
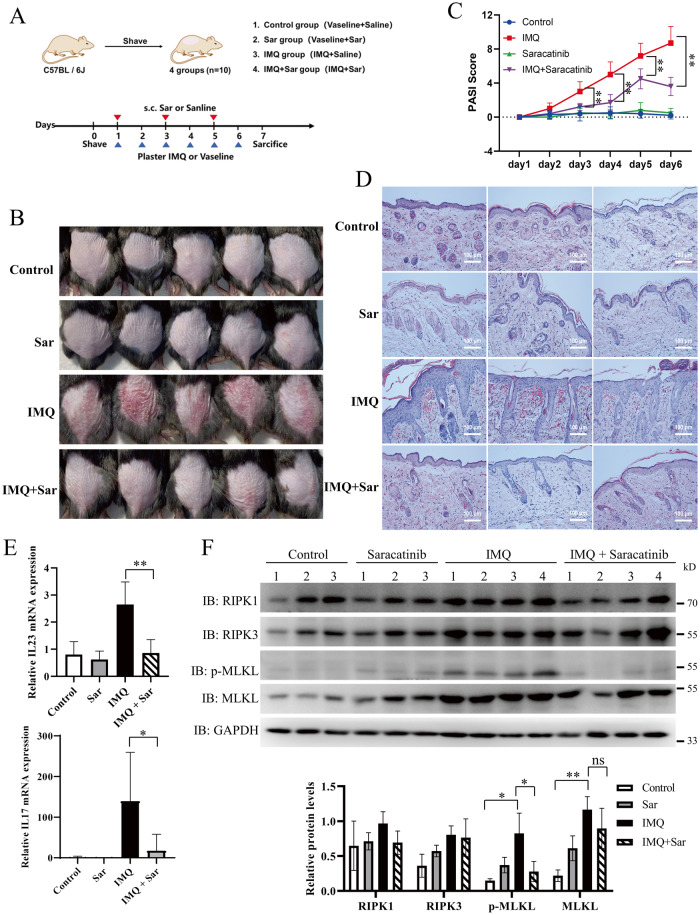
Saracatinib alleviates IMQ-induced psoriasiform dermatitis. **A** Schematic representation of the mice experiments for the Control, Saracatinib, IMQ and IMQ + Saracatinib groups (*n* = 10). Saracatinib: 20 mg/kg. **B** Representative images of the mice dorsal on day 7 (*n* = 10). **C** Daily quantitative records of PASI score (epidermal erythema, thickness, and scales) of mice dorsal skin (*n* = 10). **D** Representative H&E staining images of cross-sectional slices of the mice dorsal on day 7 (*n* = 9). Scale bar = 100 µm. **E** IL-23 and IL-17 mRNA expression levels in tissues were determined by quantitative real-time PCR analysis (*n* = 8). (**F**) The relative skin tissues were lysed. Protein levels of RIPK1, RIPK3, MLKL, p-MLKL, and GAPDH were determined by western blotting (*n* = 3 for control and saracatinib, *n* = 4 for IMQ and IMQ + Saracatinib). The intensities of the indicated protein expression levels were quantified by densitometry and plotted. *: *p* < 0.05, **: *p* < 0.01. Data are represented as mean ± SD. See also Figs. S9–S11.

Necroptosis has been implicated in psoriatic inflammation and IMQ-induced psoriasis mouse model [21–26]. Plasma membrane rupture is one of the hallmarks of necroptosis, which released endogenous molecules including high mobility group box 1 (HMGB1) and heat shock protein 90 (HSP90) [37]. Importantly, both HMGB1 and HSP90 were considered as damage-associated molecular patterns (DAMPs) and implicated in psoriatic skin inflammation [38–40]. As shown in Fig. S8A, B, saracatinib inhibited the release of HMGB1 and HSP90 induced by TNF-induced necroptosis. To investigate the therapeutic potential of saracatinib in vivo, we examined the effect of saracatinib on IMQ-induced psoriasiform dermatitis in mice and found that saracatinib markedly ameliorated psoriatic lesions in mice (Fig. 6A, B). Compared with the control group, IMQ cream caused erythema, thickening and scaling on the dorsal skin of mice. In contrast, saracatinib markedly alleviated the symptoms induced by IMQ (Fig. 6B). Consistently, PASI scores were significantly lower in the IMQ + saracatinib group than in the IMQ group (Figs. 6C and S9A–C). Saracatinib alone had no obvious effect on the dorsal skin of mice. Psoriasis can induce a systemic inflammatory response in mice, leading to significant spleen enlargement [41, 42]. Therefore, we also evaluated the spleen size and weight of mice in different treatment groups and found that IMQ led to spleen enlargement, while saracatinib treatment significantly inhibited this change (Fig. S10A, B). Moreover, saracatinib markedly alleviated IMQ-induced psoriasis-like parakeratosis, hyperkeratosis, epidermal thickening, and inflammatory cell infiltration in mouse skin tissues (Figs. 6D, S11A, B). Interleukin-17 (IL-17) and IL-23 are closely associated with the onset and development of psoriasis [43, 44]. Quantitative real-time PCR results showed that saracatinib significantly reduced the expression of IL-17 and IL-23 in mouse skin tissues (Fig. 6E). Phosphorylation of MLKL is the hallmark of necroptosis. Consistent with a previous study [26], IMQ induced the phosphorylation of MLKL in mouse skin tissues (Fig. 6F). The phosphorylation of MLKL was significantly reduced in the IMQ + saracatinib group compared with the IMQ group (Fig. 6F). Taken together, these results indicate that saracatinib alleviated IMQ-induced psoriasis by inhibiting the phosphorylation of MLKL.

## Discussion

Since necroptosis has been implicated in multiple pathological diseases, the development of necroptosis inhibitors are potential therapeutic agents for the treatment of necroptosis-related diseases. Many necroptosis inhibitors, most of which are inhibitors of RIPK1 and RIPK3, have been shown to protect cells from necroptosis in vitro and in vivo [45–48]. As MLKL is the executor of necroptosis, MLKL inhibitors have attracted great attention. Several MLKL inhibitors have been discovered. Compound GW806742X was found to inhibit necroptosis by binding to the nucleotide-binding site within the MLKL pseudokinase domain [49]. However, GW806742X might also inhibit the kinase activities of RIPK1 and RIPK3, which raises the question of whether GW806742X-mediated inhibition of necroptosis is completely dependent on the targeting of MLKL [50]. Several covalent inhibitors of MLKL, including necrosulfonamide (NSA), TC13172, compounds 56 and 66, and their derivatives, have also been developed [9, 50, 51]. These inhibitors covalently bind to the Cys86 residue of human MLKL and inhibit the translocation of MLKL to the cell membrane. However, since Cys86 is absent in the mouse MLKL protein, these covalent inhibitors fail to inhibit necroptosis in mouse cells, which limits the usage of these inhibitors in mouse models. Our work revealed that saracatinib did not inhibit the activation, interaction, and translocation of RIPK1 or RIPK3 induced by TNF, which indicated that saracatinib plays roles in signalling downstream of RIPK1 or RIPK3. Saracatinib bound to MLKL and interfered with the phosphorylation, oligomerization, and plasma membrane translocation of MLKL. Consistently, MLKL Q343A attenuated the inhibitory effect of saracatinib on TNF-induced necroptosis. Moreover, the saracatinib-binding sites of MLKL are highly conserved among species, which could explain why saracatinib inhibited TNF-induced necroptosis in both human and mouse cell lines. We believe that saracatinib could be a useful tool to investigate the function of MLKL in mouse models.

Saracatinib was originally discovered as a tyrosine kinase inhibitor [52] and has been used in several failed clinical trials of various tumours. Recent studies have shown that saracatinib is able to cross the blood-brain barrier and alleviate neuropathic diseases in animal models, and the compound has been used in clinical trials for Alzheimer’s disease (AD) [28, 53–55]. Accumulated evidence indicates that necroptosis plays roles in neurodegenerative diseases [56, 57]. According to our work, saracatinib was able to inhibit necroptosis, and it would be interesting to know whether saracatinib influences neuropathic diseases by regulating necroptosis. We noticed that saracatinib induced cell death at a high dose, which implied that there might be some side effects associated with the use of saracatinib. Although clinical trials have shown that saracatinib is generally safe and well tolerated in patients with mild-to-moderate AD [28], the efficacy and side effects of saracatinib in the clinical treatment of psoriasis still need to be studied.

Psoriasis is a common chronic inflammatory and autoimmune skin disease that affects more than 100 million people worldwide [58]. The histopathological changes that occur in psoriasis include acanthosis, parakeratosis, thinning of the granular layer, dilated capillaries, and neutrophil infiltration. Many factors, such as genetic factors, the immune response and environmental stimuli, promote disease progression [43, 44]. However, the detailed pathogenesis of psoriasis remains unclear. Previous reports have shown that necroptosis plays an important role in the onset and development of psoriasis, and inhibition of the necroptosis pathway alleviates the development of psoriasis [21–24]. Importantly, the hallmarks of necroptosis, such as phosphorylation of RIPK3 and MLKL were significantly increased in the epidermis of human psoriasis lesions [26]. Our work revealed that saracatinib protected mice from IMQ-induced psoriasis. Saracatinib attenuated IMQ-induced psoriasis-like parakeratosis, hyperkeratosis, acanthosis, inflammatory cell infiltration, and inflammatory cytokine expression. Moreover, saracatinib decreased IMQ-induced MLKL phosphorylation in mouse skin tissues, which implied that saracatinib ameliorated IMQ-induced psoriasis by inhibiting MLKL. We believe that our work sheds new light on how to treat necroptosis-induced pathological changes in psoriasis.

## Materials and Methods

### Antibodies and reagents

Rabbit anti-Flag (20543-1-ap), mouse anti-GAPDH (60004-1-Ig), goat anti-mouse (SA00001-1), and goat anti-Rat (SA00001-15) antibodies were purchased from Proteintech (USA). Goat antirabbit (A5014) antibodies were purchased from ABclonal (USA). Rabbit anti-JNK (9252T), p-JNK (T183/Y185) (4668T), p-IκB (2859T), p-mRIPK1 (S166) (53286S), p-mRIPK1 (S321) (38662S), hRIPK1 (3493T), hRIPK3 (13526S), hMLKL (14993S), p-hRIPK1 (S166) (65746S), p-hRIPK3 (S227) (93654S), p-hMLKL(S358) (91689S), and mouse anti-IκB (4814T) were purchased from Cell Signaling Technology (USA). Rabbit anti-Vimentin (ab92547), p-mRIPK3 (S232/T231) (ab222320), p-mMLKL (S345) (ab196436), and rat anti-mMLKL (ab243142) were purchased from Abcam (England). Mouse anti-mRIPK1 (610458) was purchased from BD Biosciences (USA). DiscoveryProbe™ FDA-approved Drug Library (96-well) was obtained from Apexbio (USA). Saracatinib, z-VAD, Smac mimetic (SM-164), Necrostatin-1, GSK872 were obtained from TargetMol. 4-Hydroxytamoxifen (4-OHT) was obtained from MCE. TNF-α and IFN-γ were obtained form novoprotein.

### Cell culture

The L929 cell line, HEK293T cell line, NIH3T3-RIPK3 cell line, HT-29 cell line, Hela-RIPK3 cell line, and Mouse RIPK3-flag reconstituted RIPK3-KO L929 cell line were kindly provided by Professor Jiahuai Han (Xiamen University, Xiamen). The mouse fibrosarcoma L929 cell line, HEK293T cell line, NIH3T3-RIPK3 cell line and Hela-RIPK3 cell line were maintained in Dulbecco’s Modified Eagle’s Medium (DMEM). The human colon cancer HT-29 cell line was maintained in RPMI-1640 medium. All of cell lines were supplemented with 10% foetal bovine serum, 4 mM L-glutamine, 100 IU penicillin and 100 mg/mL streptomycin at 37 °C in a humidified incubator containing 5% CO2. All of the cell lines have been authenticated using STR profiling and no mycoplasma contamination was detected.

### Cell death analysis

Cell death was monitored using the CCK8 kit (MCE, USA) according to the manufacturer’s instructions. Cells were seeded in 96-well plates and treated as indicated. 10 μL CCK8 reagent was added to each well and incubated for 1 h before measuring the optical absorbance at 450 nm using a microplate reader (Eppendorf, Germany). For PI assay, cells were seeded in 24-well plates and treated as indicated. Cells were incubated with PI (1ug/ml) for 5 min, and fluorescence intensity was detected by flow cytometry (BD, USA) or photographed.

### Artificially induced dimerization/oligomerization of proteins in the necroptosis signalling pathway

Plasmids expressed TNFR1 death domain-HBD, RIPK1-HBD, RIPK3-HBD, or MLKL-1-190-HBD were kindly provided by prof. Jiahuai Han (Xiamen University, Xiamen) [32]. Artificially induced dimerization/oligomerization of TNFR1 death domain-HBD, RIPK1-HBD, RIPK3-HBD, or MLKL-1-190-HBD were induced by 4-hydroxytamoxifen (4-OHT) [59]. L929 cells stably expressing TNFR1 death domain-HBD, RIPK1-HBD, RIPK3-HBD, or MLKL-1-190-HBD were treated 4-OHT + z-VAD to induced necroptosis. The RIPK1 kinase inhibitor Nec-1 and RIPK3 kinase inhibitor GSK872 were chose as control compound.

### Immunoprecipitation and immunoblotting

Cells were seeded into 10 cm cell culture dishes and treated as indicated. After washing with cold PBS, cells were harvested with lysis buffer (20 mM Tris-HCl pH 7.5, 120 mM NaCl, 1 mM EDTA, 1 mM EGTA, 1% Triton X-100, 2.5 mM Sodium pyrophosphate, 1 mM β-Glycerophosphate, 1 mM Na3VO4, 1 mM PMSF, Protease inhibitor cocktail (TargetMol)). The samples were sonicated and centrifuged at 4 °C 12,000 rpm for 30 min. 100ul of the supernatant was added to an equal volume of 2×SDS loading buffer and then detected by Western blot. The remaining supernatant was incubated with prewashed M2 (anti-Flag) beads overnight at 4 °C. The beads were washed four times with lysis buffer and then boiled in 100 μL of 1× SDS loading buffer for 10 min. Samples were loaded on SDS-PAGE gels, followed by electroblotting onto PVDF membranes (Millipore) and immunoblotting with the indicated antibodies.

### Molecular docking

Obtain the molecular structure of saracatinib from the PubChem compound database (https://pubchem.ncbi.nlm.nih.gov/) and retrieve the protein structure of MLKL from the PDB database (http://www.rcsb.org/) with PDB ID 4BTF (resolution: 2.60 Å) [35]. Convert all files to PDBQT format, remove all water molecules, and add polar hydrogen atoms. Center the grid box and allow for free molecular movement. Set the docking pocket to a cubic space of 30 Å × 30 Å × 30 Å, with a grid spacing of 0.05 nm. Molecular docking studies were performed using Autodock Vina 1.2.2 (http://autodock.scripps.edu/) for model visualization [60].

### MLKL oligomerization analysis

Cells were seeded in 6-well plates and treated as indicated. After washing with cold PBS, cells were harvested with loading buffer without β-mercaptoethanol (50 mM Tris-Hcl pH6.8, 2% SDS, 10% glycerin, 12.5 mM EDTA, 0.02% bromophenol). Samples were sonicated and loaded on 8% SDS-PAGE gels followed by electroblotting onto PVDF membrane. and then probed with the indicated antibodies.

### Extraction of detergent-insoluble fraction

Cells were seeded in 6 cm cell culture dishes and treated as indicated. After washing with cold PBS, the cells were harvested with lysis buffer. Samples were centrifuged at 500 g for 5 min, the precipitate was discarded, and the supernatant was centrifuged at 12,000 rpm for another 5 min. The precipitate is the insoluble fraction, which was washed with lysis buffer and an appropriate amount of 1×SDS loading buffer was added. The samples were then sonicated and detected by western blot.

### Mice

Specific pathogen-free C57BL/6 mice were obtained from GemPharmatech Co.,Ltd. (Nanjing, China). 6 to 8-week-old male C57BL/6 mice weighing 18–22 g were maintained at a constant temperature under a 12-h light-dark cycle with unrestricted access to standard chow and water. All animal experiments were performed in accordance with protocols approved by Institutional Animal Ethical Committee of Fujian Medical University.

### IMQ-induced model of psoriasiform dermatitis

C57BL/6 mice were randomly divided into several groups (*n* = 10). On the second day after dorsal hair removal, Petroleum jelly was applied to the control group and saracatinib group, and 50 mg 5% IMQ cream (Med-Shine Pharmaceutical, Sichuan, China) was applied to the IMQ group and IMQ + saracatinib group on the shaved dorsal skin for 6 consecutive days. The control group and IMQ group were injected subcutaneously with saline, and the saracatinib group and IMQ + saracatinib group were injected subcutaneously with saracatinib (20 mg/kg/day) every other day before IMQ or petroleum jelly application. Mice were euthanized on day 7. The Psoriasis Area and Severity Index (PASI) scores of the dorsal skin of the mice were evaluated daily to assess their clinical changes. Skin tissues were collected for subsequent studies. Spleens were collected for photographs and weight statistics.

### Quantitative real-time RT-PCR

RNA extraction of tissues was performed using Trizol as previously described [61]. The extracted RNA was reverse transcribed into cDNA using TransScript Uni All-in-One First-Strand cDNA Synthesis SuperMix (TransGen Biotech) according to the instructions. Quantitative real-time PCR based on PerfectStart Green qPCR SuperMix(TransGen Biotech) was performed using the AriaMx Real-time PCR System (Agilent, USA). Relative gene expression was normalized to β-actin and calculated by the 2-ΔΔCt method.

### Cellular thermal shift assay (CETSA)

Cells were seeded into 6 cm cell culture dishes and treated with or without saracatinib for 3 h. After washing with cold PBS, the cells were harvested with a cell scraper and divided into several tubes, incubated at different temperatures (from 37 °C to 67 °C) for 3 min, cooled at room temperature for 3 min, frozen and thawed repeatedly for three times, and then centrifuged at 4 °C and 12,000 rpm for 20 min to collect the supernatant. After mixing with loading buffer and denaturing at 100 °C for 10 min, western blotting was used for analysis.

### Immunofluorescence assay

Cells were seeded onto BeyoGold™ 35 mm confocal slides and treated as indicated. After three washes with cold PBS, samples were fixed with 4% paraformaldehyde for 15 min, washed three times with PBS, fixed with 0. 5% Triton X-100 for 15 min, washed three times with PBS, incubated with 5% bovine serum albumin for 1 h at room temperature, incubated with appropriate antibodies overnight at 4 °C, washed four times with PBST, FITC-labeled fluorescent secondary antibody was added and incubated for 30 min at room temperature, followed by PBST wash three times, DAPI was used to stain the nucleus for 5 min, PBST wash four times, and a LEICA SP5 confocal laser microscope (Leica, Germany) was used for photography.

### Hematoxylin and eosin (HE) staining

HE staining was performed according to a routine protocol. Briefly, paraffin sections of mouse dorsal tissues were prepared by xylene dewaxing and ethanol gradient hydration, followed by hematoxylin solution for 5 min, 1% acid ethanol (1% HCl in 75% ethanol) differentiation for a few seconds, 0.5% ammonia water for 2 min, and eosin staining for 3 min. The sections were then dehydrated through an ethanol gradient and made transparent with xylene. After sealing in neutral rubber, the sections were examined and photographed using the LEICA DM2700M (Leica, Germany).

### Statistical Analysis

Data were analyzed with GraphPad Prism 9. *T*-tests was used to compare differences between columns. Data are presented as mean ± standard deviation (SD). All experiments were performed in at least triplicate. Statistical significance was defined as *p* < 0.05. *p < 0.05, **p < 0.01.

### Supplementary information

Supplemetal Figures

aj-checklist

## Data Availability

All datasets generated and analysed during this study are included in this published article and its Supplementary Information files. Additional data are available from the corresponding author on reasonable request.
