# Supplementary material for: Saracatinib inhibits necroptosis and ameliorates psoriatic inflammation by targeting MLKL
Source: Cell Death Dis. 2024 Feb 8;15(2):122. doi: 10.1038/s41419-024-06514-y (PMC10853205; doi:10.1038/s41419-024-06514-y)
Supplement: Supplementary file 1 — Supplemetal Figures [file 41419_2024_6514_MOESM1_ESM.pdf]

Figure S1

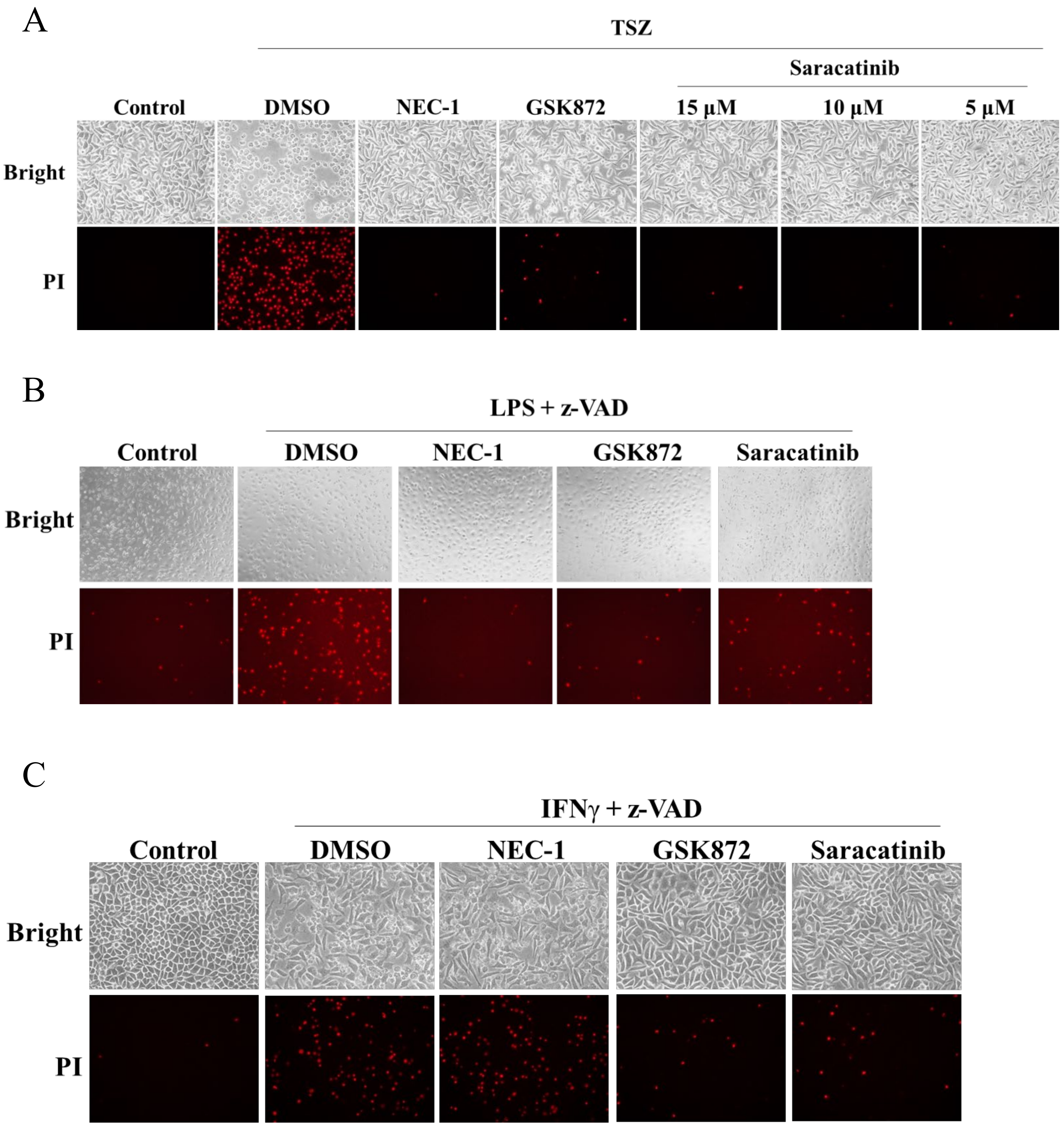

Figure S1. Saracatinib inhibited necroptosis in different cell lines. Related to Figure 1. (A) L929 cells were pretreated with NEC-1 (20 µM), GSK872 (10 µM), saracatinib (5 µM, 10 µM, and 15 µM) for 1 hour following treatment with TSZ to induce necroptosis. Then the cells were incubated with propidium iodide (PI). Representative photographs were taken with a fluorescence microscope. (B) Similar to (A) except that BMDM cells were pretreated with NEC-1 (20 µM), GSK872 (10 µM), saracatinib (15 µM), for 1 hour following treatment with LPS + z-VAD to induce necroptosis. Related to Figure 1E. (C) Similar to (A) except that RIPK1-KO L929 cells were pretreated with NEC-1 (20 µM), GSK872 (10 µM), saracatinib (15 µM), for 1 hour following treatment with IFN-γ+ z-VAD to induce necroptosis. Related to Figure 1F.

Figure S2

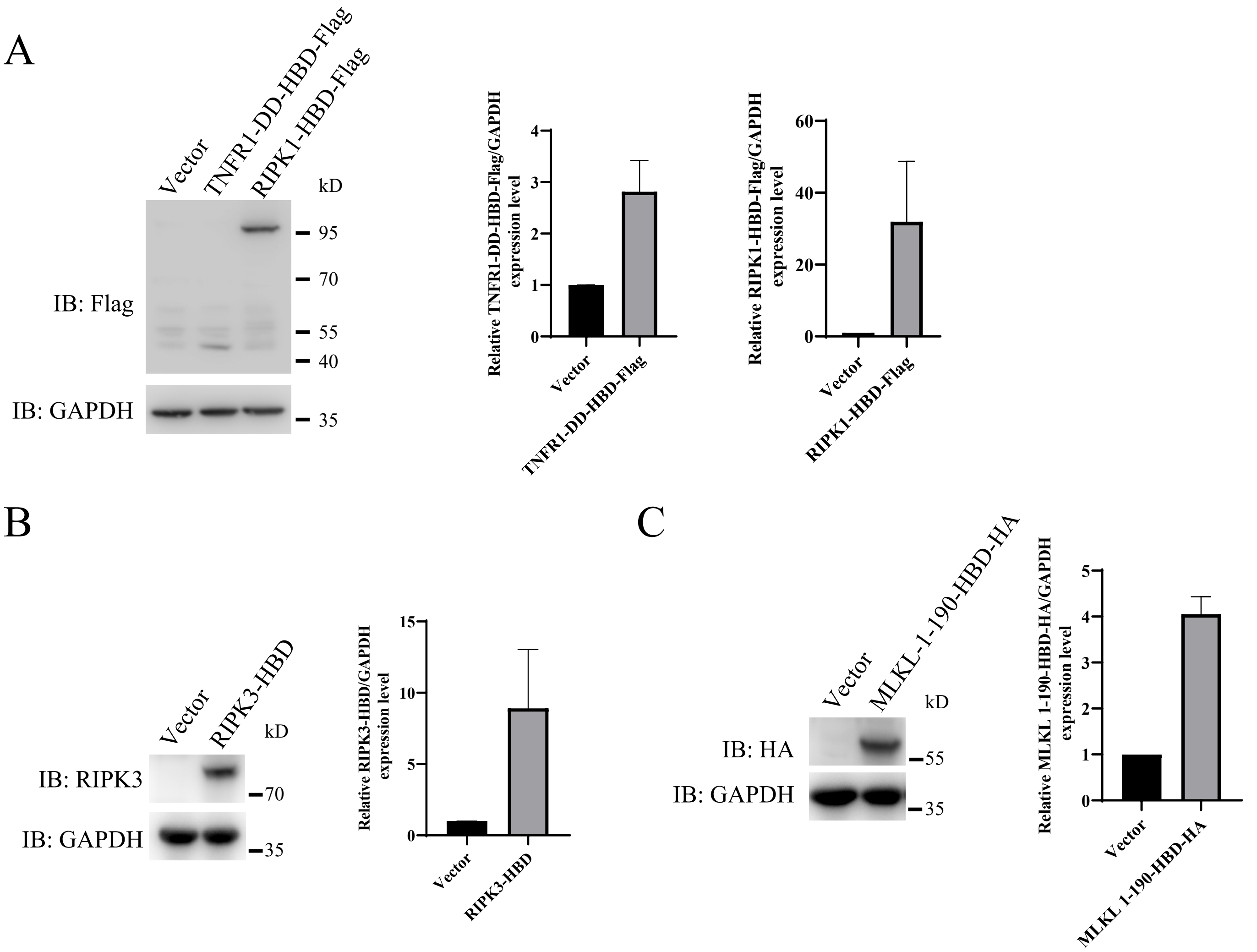

Figure S2. The protein expression level of TNFR1-death domain (DD), RIPK1, RIPK3, and MLKL-N-terminal domain fused with HBD. Related to Figure 2. L929 cells were infected with lentivirus expressing vector, TNFR1-DD-HBD-Flag (A), RIPK1-HBD-Flag (A), RIPK3-HBD (B) or MLKL-1-190-HBD-HA (C). Then the cells were harvested and analyzed with the indicated antibodies (n=3).

Figure S3

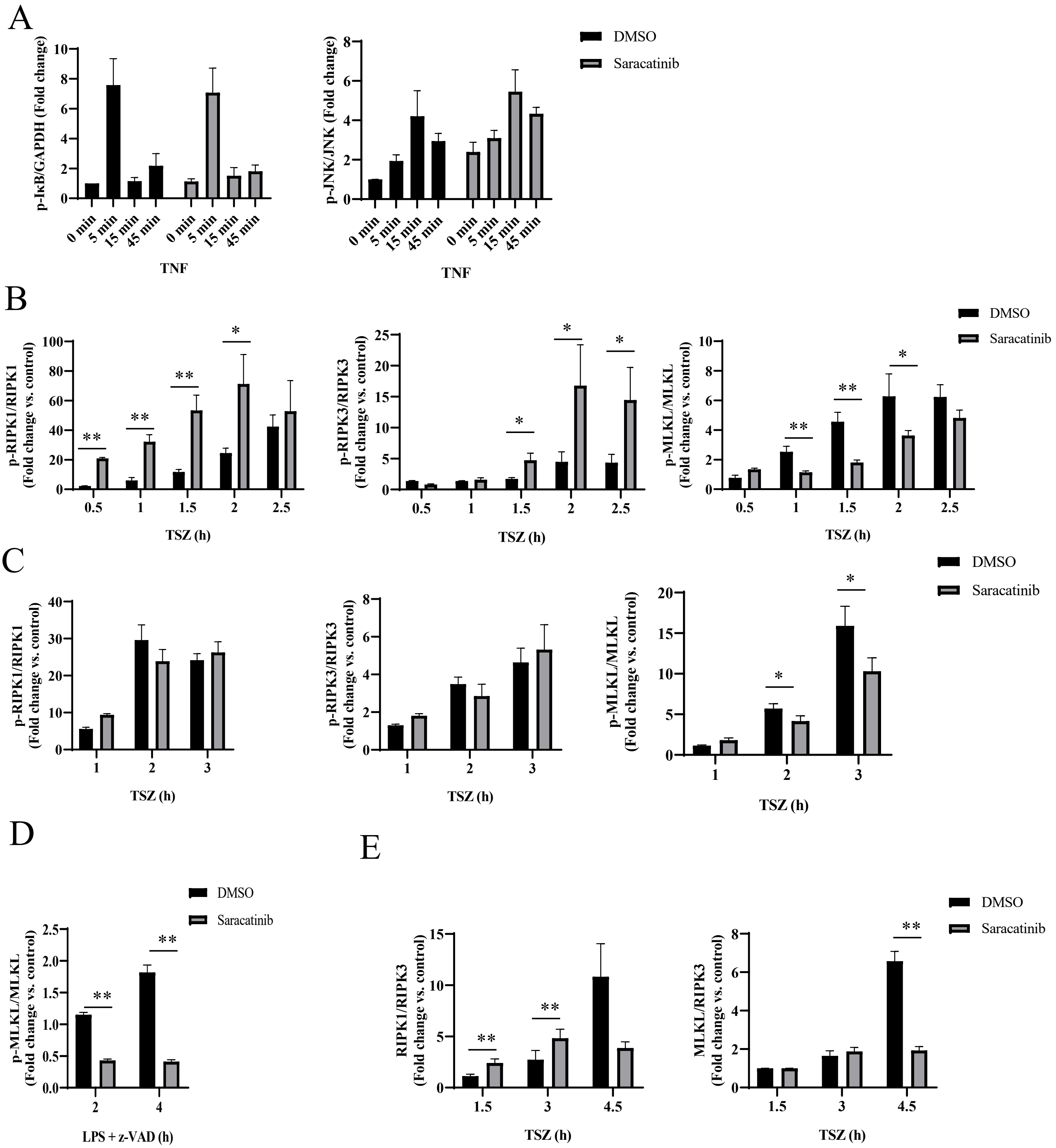

Figure S3. The protein densitometry analysis of three independent biological replicates. Related to Figure 3. (A) Graphs represent the quantification of the blots related to Figure 3A. (B) Graphs represent the quantification of the blots related to Figure 3B. (C) Graphs represent the quantification of the blots related to Figure 3C. (D) Graphs represent the quantification of the blots related to Figure 3D. (E) Graphs represent the quantification of the blots related to Figure 3E. *: $p < 0.05$, **: $p < 0.01$. Data are represented as mean ± SD.

Figure S4

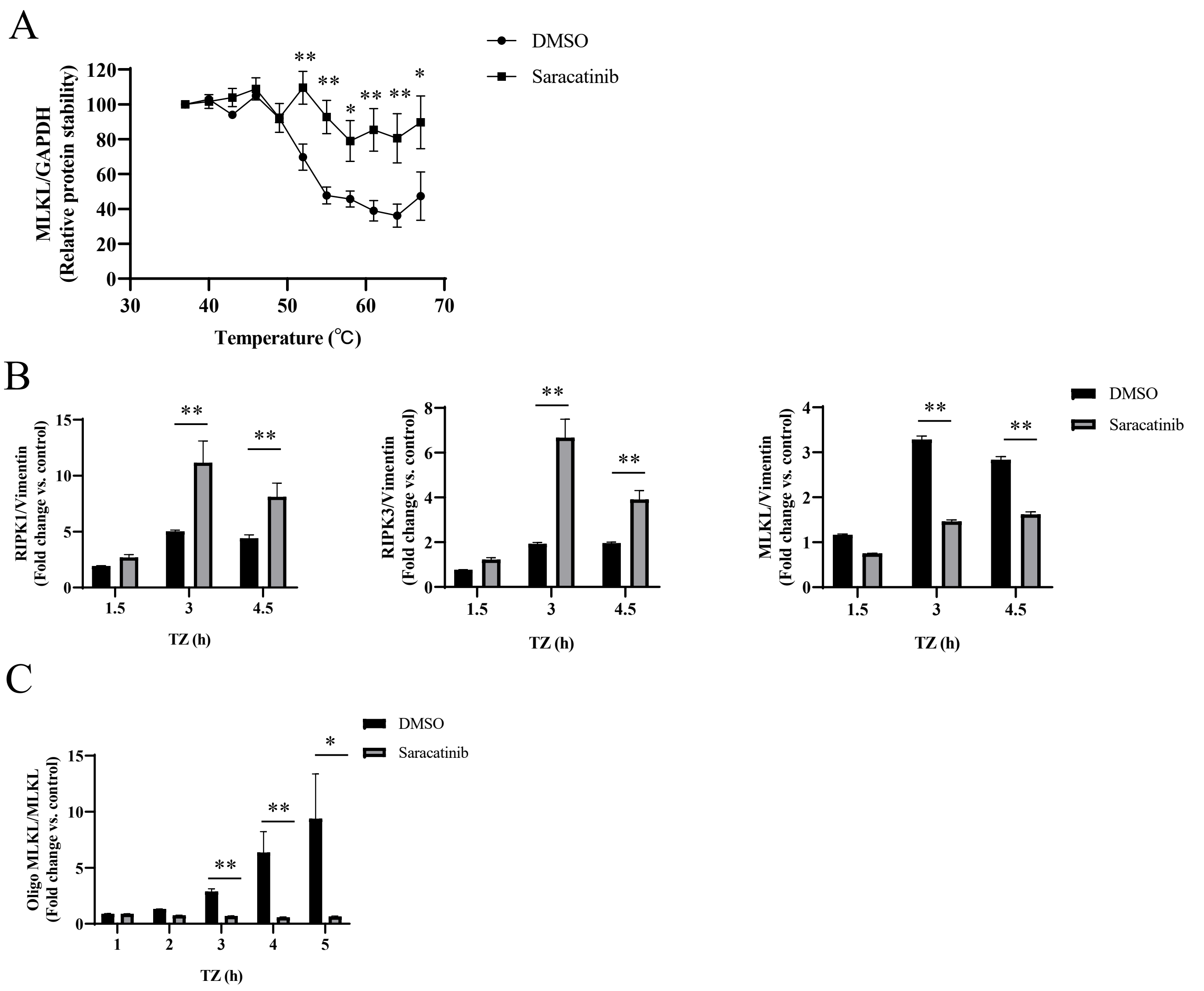

Figure S4. The protein densitometry analysis of three independent biological replicates. Related to Figure 4. (A) Graphs represent the quantification of the blots related to Figure 4C. (B) Graphs represent the quantification of the blots related to Figure 4D. (C) Graphs represent the quantification of the blots related to Figure 4E. *: $p < 0.05$, **: $p < 0.01$. Data are represented as mean ± SD.

Figure S5

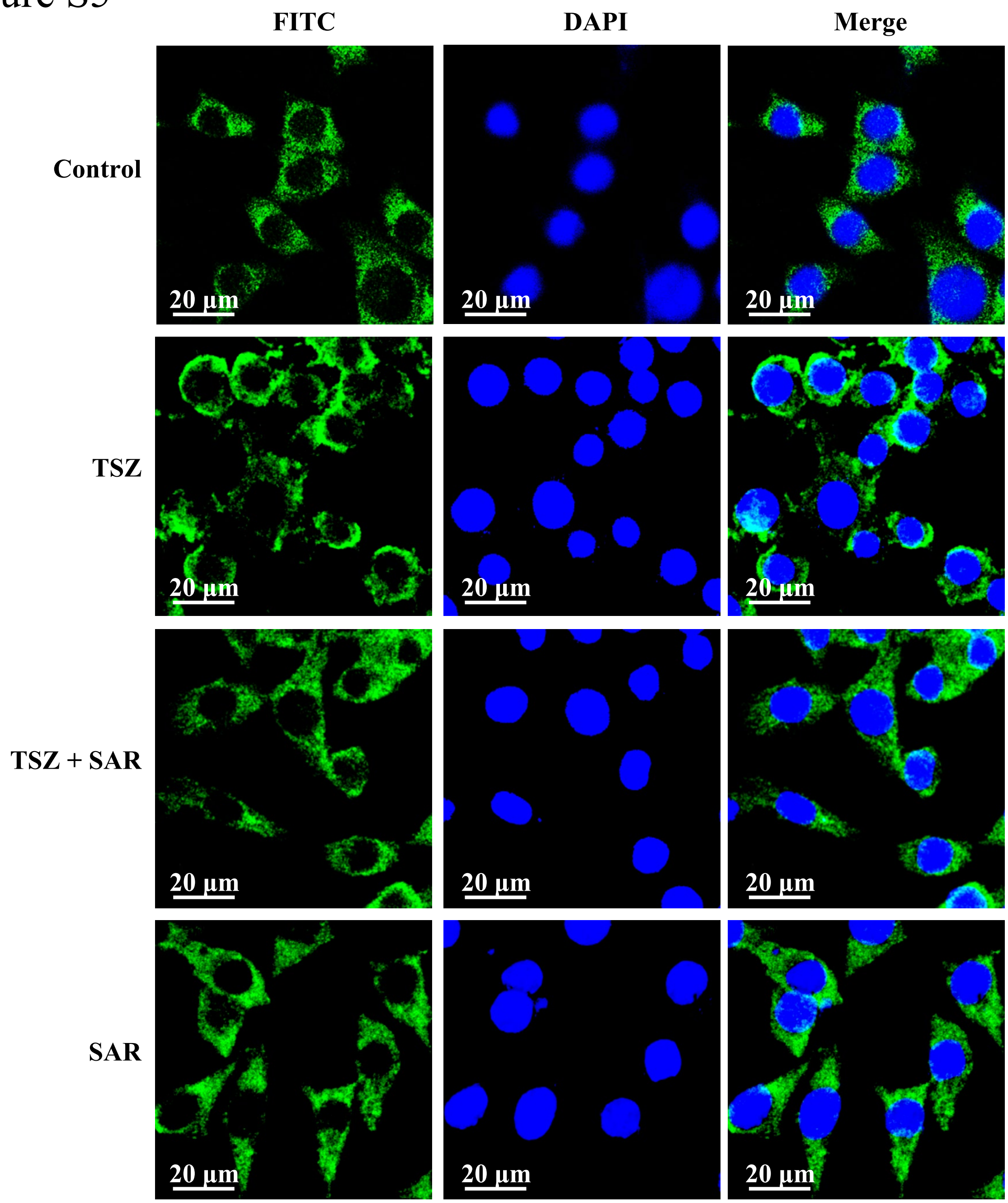

Figure S5. saracatinib inhibit plasma membrane translocation of MLKL. L929 cells were pretreated saracatinib (7.5 μM) for 1 hour following treatment with TSZ to induce necroptosis. MLKL (Green) was immunostained with MLKL primary antibodies and , FITC-labeled secondary antibody. DAPI (Blue) is used for nuclear staining.

Figure S6

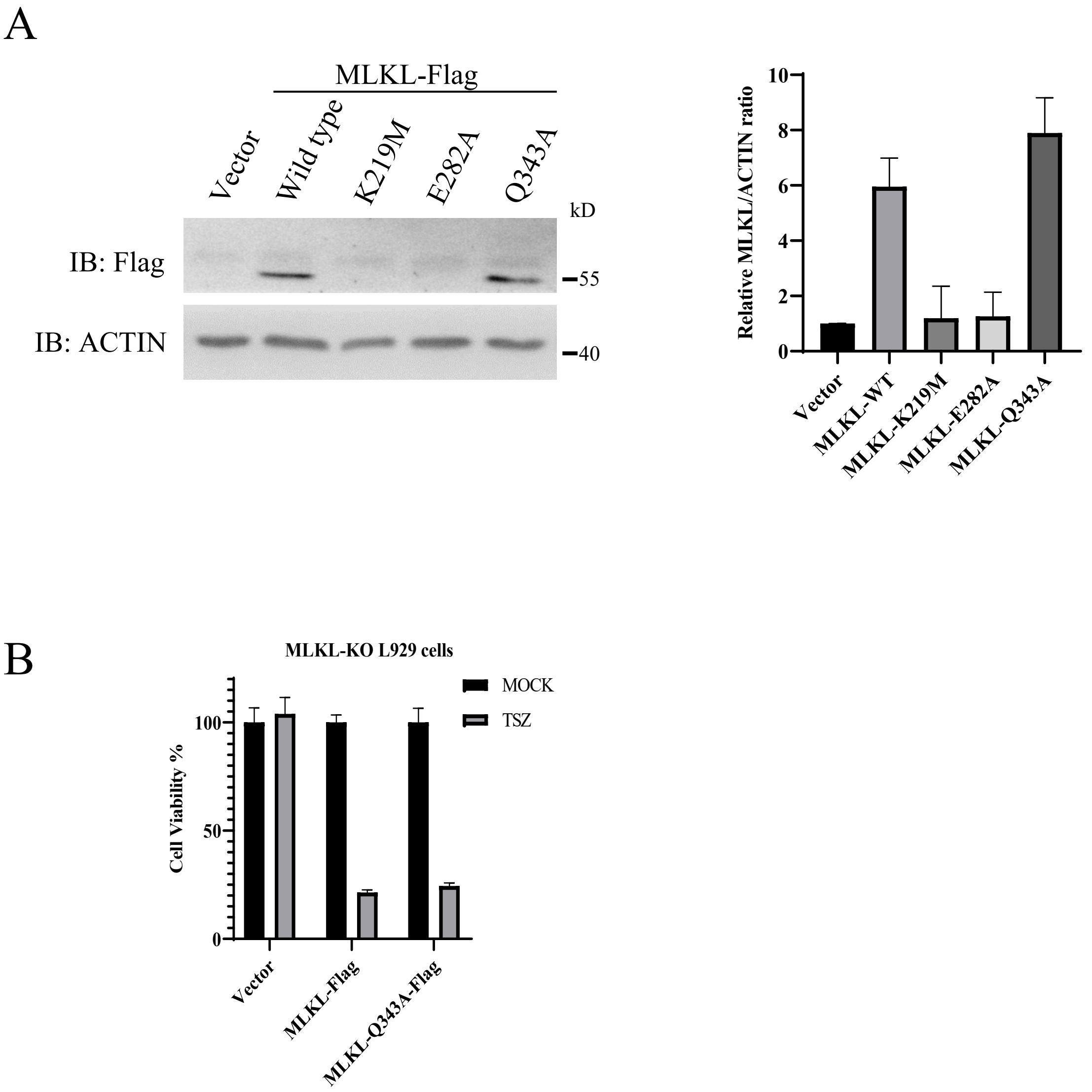

Figure S6. Reconstitution of MLKL-KO L929 cells with vector, MLKL or its mutants. (A) MLKL-KO L929 cells were infected with lentivirus expressing empty vector, MLKL or its mutants to generate stable cell lines. Then the cells were harvested and analyzed with the indicated antibodies (n=3). (B) MLKL-KO L929 cells expressing empty vector, MLKL or MLKL-Q343A were treated with or without TSZ (n=4). Then the cell viabilities were determined by CCK8 method.

Figure S7

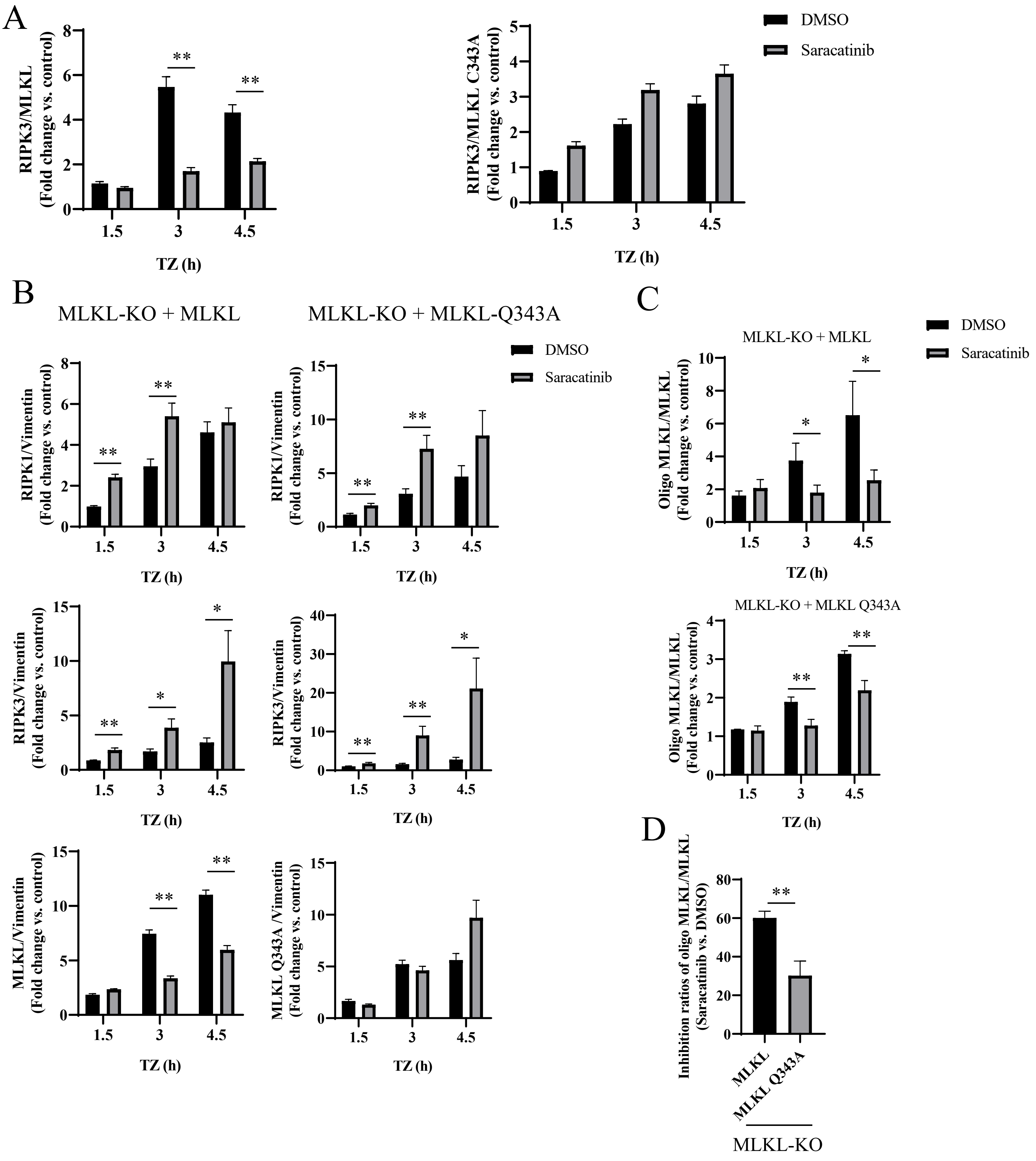

Figure S7. The protein densitometry analysis of three independent biological replicates. Related to Figure 5. (A) Graphs represent the quantification of the blots related to Figure 5B. (B) Graphs represent the quantification of the blots related to Figure 5C. (C) Graphs represent the quantification of the blots related to Figure 5D. (D) Inhibition ratios of oligo MLKL/MLKL (Saracatinib vs. DMSO) when MLKL-Flag or MLKL-Q343A-Flag reconstituted MLKL-KO L929 cells were treated with TZ for 4.5 hours. Related to Figure 5D. *: $p < 0.05$, **: $p < 0.01$. Data are represented as mean ± SD.

Figure S8

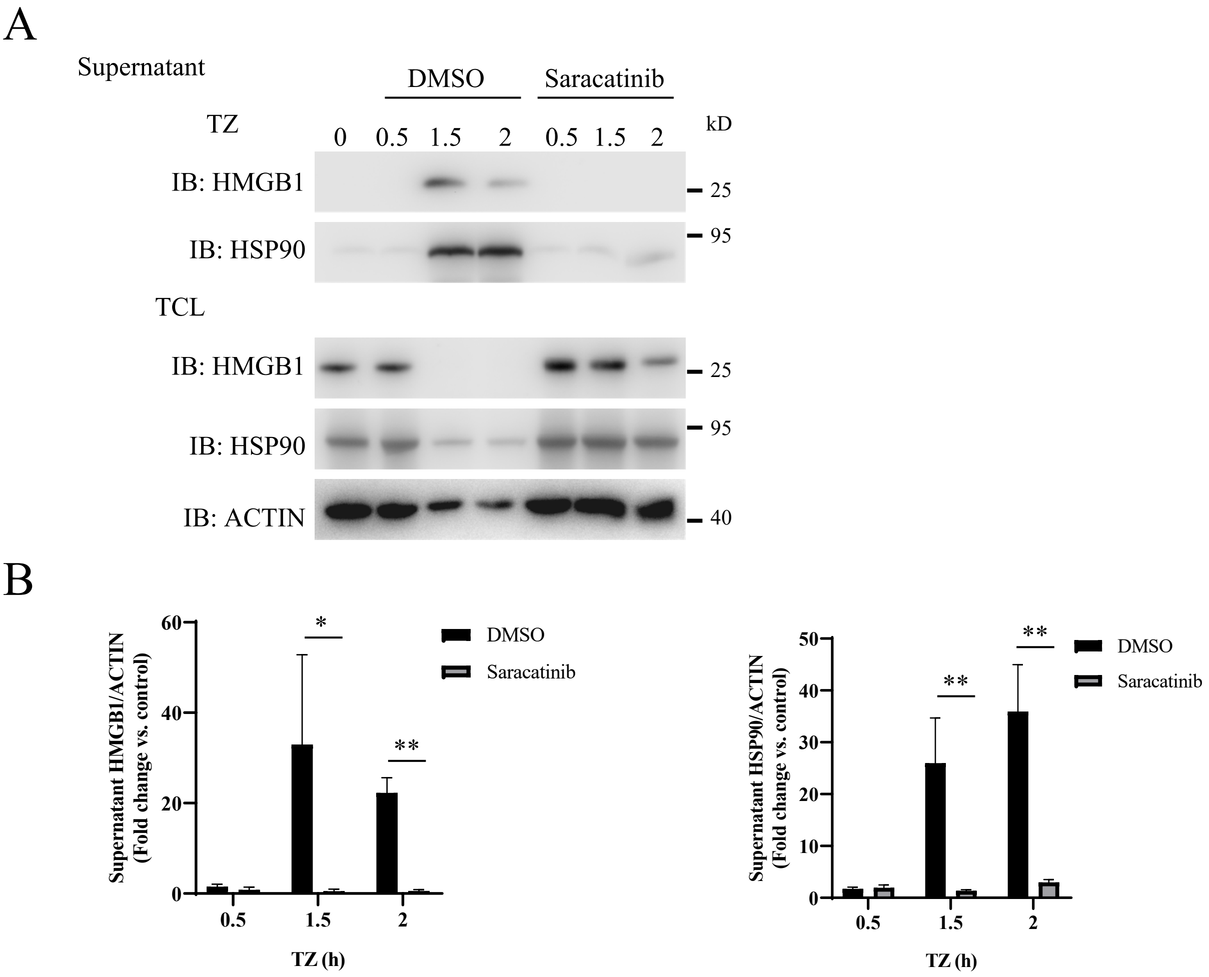

Figure S8. Saracatinib inhibited necroptosis mediated release of HMGB1 and HSP90. (A) L929 cells were pretreated with saracatinib (7.5 μM) for 1 hour following treatment with TZ for indicated time. Then the cells and supernatants were harvested and analyzed with the indicated antibodies (n=3). (B) Graphs represent the quantification of the blots related to Figure S8A. *: $p < 0.05$, **: $p < 0.01$. Data are represented as mean ± SD.

Figure S9

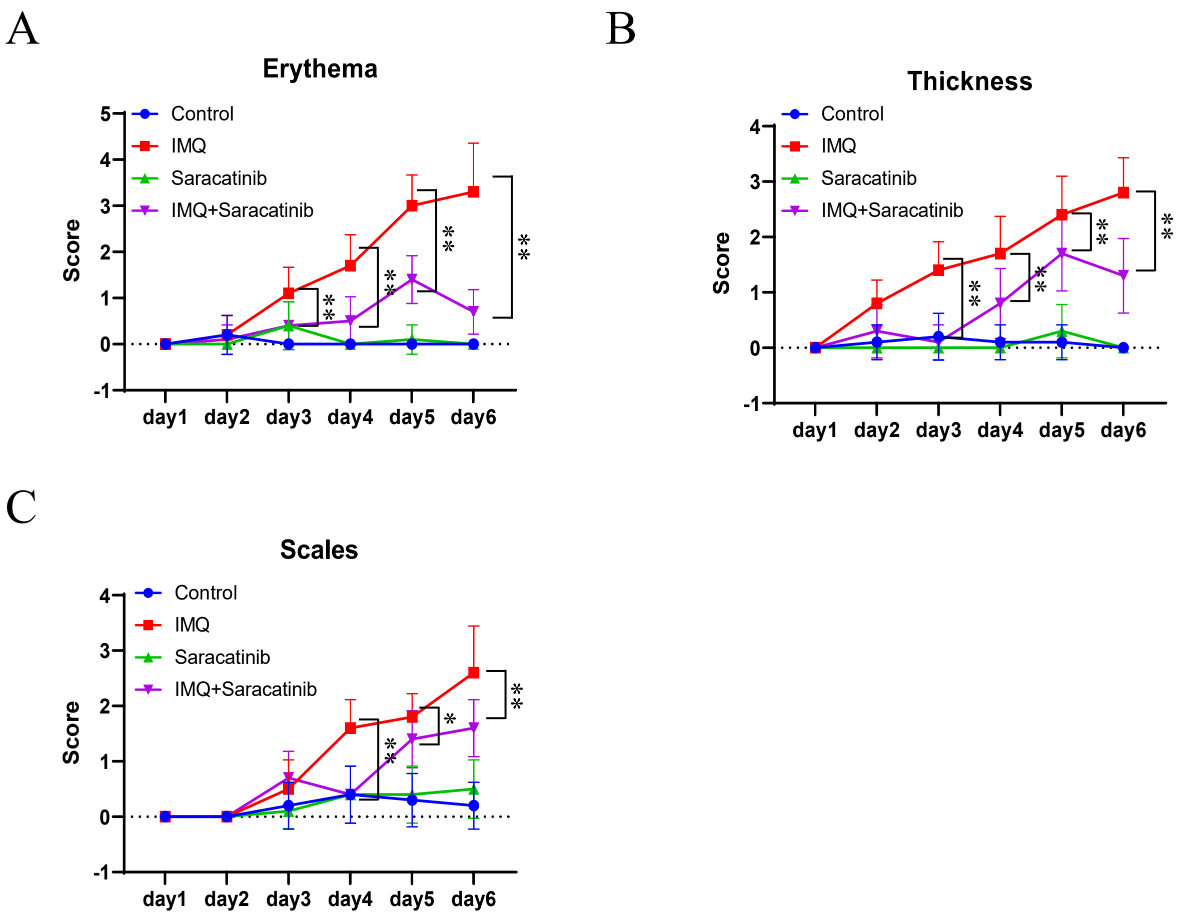

Figure S9. The detail of PASI score of IMQ-induced mice psoriasis treated with or without saracatinib. (A)-(C) Daily quantitative records of epidermal erythema (A), thickness (B), and scales (C) score of mice dorsal skin (n=10). *: $p < 0.05$, **: $p < 0.01$. Data are represented as mean ± SD.

Figure S10

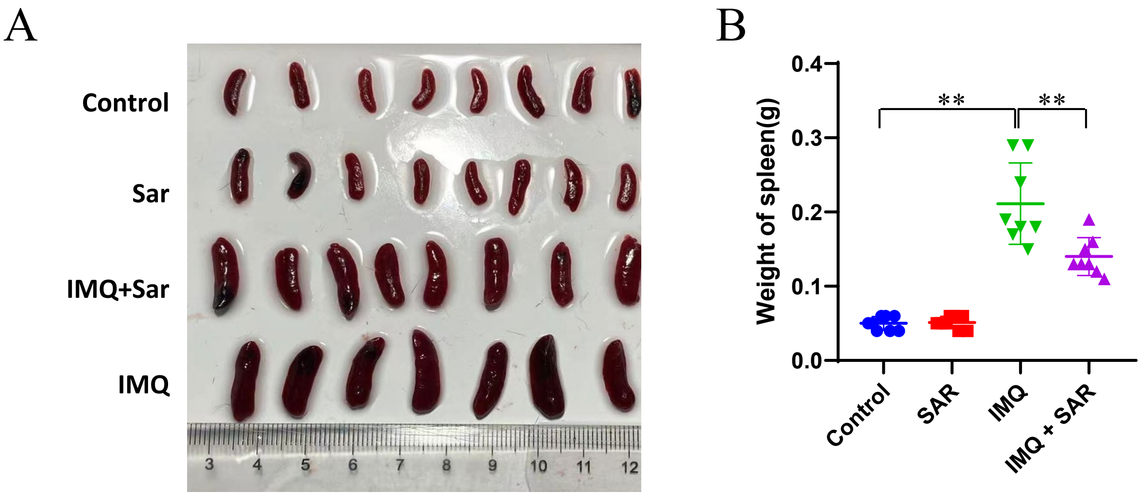

Figure S10. The spleen of IMQ-induced mice psoriasis treated with or without saracatinib on day 7. Images of indicated mice spleen (A) and related spleen weight (B) (n=8). **: $p < 0.01$. Data are represented as mean ± SD.

Figure S11

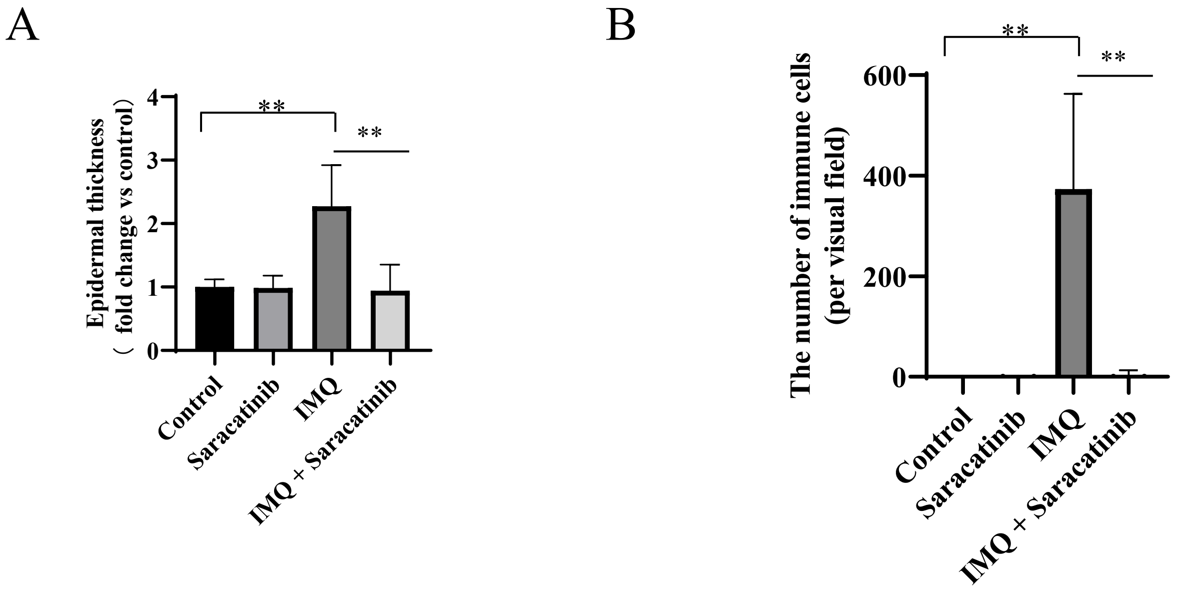

Figure S11. Saracatinib markedly alleviated IMQ-induced epidermal thickening and inflammatory cell infiltration in mouse skin tissues. Related to Fig. 6D. (A) The epidermal thickness of each visual field was calculated and normalized to control (n=9). (B) The number of immune cells of each visual field was calculated and normalized to control (n=9). **: $p < 0.01$. Data are represented as mean ± SD.
